# “Hit the Robot on the Head With This Mallet” – Making a Case for Including More Open Questions in HRI Research

**DOI:** 10.3389/frobt.2021.603510

**Published:** 2021-02-25

**Authors:** Katie A. Riddoch, Emily. S. Cross

**Affiliations:** ^1^ Social Brain in Action Laboratory, Institute of Neuroscience and Psychology, University of Glasgow, Glasgow, Scotland, United Kingdom; ^2^ Social Brain in Action Laboratory, Department of Cognitive Science, Macquarie University, Sydney, NSW, Australia

**Keywords:** social robotics, qualitative research, empathy, human—robot interaction, prosocial behaviour, social perception

## Abstract

Researchers continue to devise creative ways to explore the extent to which people perceive robots as social agents, as opposed to objects. One such approach involves asking participants to inflict ‘harm’ on a robot. Researchers are interested in the length of time between the experimenter issuing the instruction and the participant complying, and propose that relatively long periods of hesitation might reflect empathy for the robot, and perhaps even attribution of human-like qualities, such as agency and sentience. In a recent experiment, we adapted the so-called ‘hesitance to hit’ paradigm, in which participants were instructed to hit a humanoid robot on the head with a mallet. After standing up to do so (signaling intent to hit the robot), participants were stopped, and then took part in a semi-structured interview to probe their thoughts and feelings during the period of hesitation. Thematic analysis of the responses indicate that hesitation not only reflects perceived socialness, but also other factors including (but not limited to) concerns about cost, mallet disbelief, processing of the task instruction, and the influence of authority. The open-ended, free responses participants provided also offer rich insights into individual differences with regards to anthropomorphism, perceived power imbalances, and feelings of connection toward the robot. In addition to aiding understanding of *this* measurement technique and related topics regarding socialness attribution to robots, we argue that greater use of open questions can lead to exciting new research questions and interdisciplinary collaborations in the domain of social robotics.

## Introduction

Laboratory-based studies have demonstrated that in some contexts, people attribute human-like attributes to machines, including gender, personality, and intentions (Craenen et al., 2018; Bernotat et al., 2019; Marchesi et al., 2019). Weise et al. (2017) suggest that such attributions occur because we have an innate desire to understand our environment, and upon encountering unfamiliar agents, we default to what we are familiar with - the so-called human model. They propose that the application of the human model is why we incorrectly perceive the actions of machines as thoughtful, intentional, and emotional (Weise et al., 2017). Along similar lines, other researchers propose that the crossover between human- and robot-directed cognition can be explained by the “like-me” hypothesis of social perception and cognition, as originally proposed by developmental psychologists (Meltzoff and Prinz, 2002; Meltzoff, 2007). According to this theory, understanding the basic similarity between self and others forms the basis for social cognition, and we are biologically hard-wired to seek out self-other equivalence in other agents, including social robots (for a review, see Hortensius and Cross, 2018). Together, theoretical and empirical work examining the human model and the like-me hypothesis have led to the suggestion that in some situations, people are likely to perceive, interact, and connect with robots as though they are social agents, at least to a certain extent (Wiese et al., 2017; Hortensius and Cross, 2018; Hortensius et al., 2018).

To probe the extent to which we perceive robots as social agents, as opposed to objects, one approach researchers have used is to ask participants to inflict ‘harm’ on robotic agents. For example, previous experimenters have asked people to administer increasing levels of ‘electric shocks’ to a robot (Bartneck et al., 2005), turn a robot off and ‘wipe its memory’ (Bartneck et al., 2007), and hit a robotic animal with a hammer (Darling et al., 2015). The experimenter takes measures such as how many times the person strikes the robot, the number of pieces it was broken into, and the amount of time between being given the instruction and compliance (termed “hesitation”). Bartneck and colleagues (2005; 2007) suggested that minimal hesitation reflects the perception that the robot perceived as a sentient being. However, in these studies, participants were not asked whether they thought the robot appeared to be a living being, was an intentional agent, or anything else along these lines, so this suggestion was simply speculative at the time the studies initially appeared. In a later paper, Bartneck and Hu (2008) acknowledge this, stating that the assumption they made was not grounded in empirical evidence, but was instead included in an attempt to stimulate discussion (Bartneck and Hu, 2008).

An important question remains, however: when asked to hit a robot, what does hesitation *actually* reflect? What is this provocative paradigm *actually* measuring? Darling et al., 2015 demonstrated that after being asked to hit a robot bug, individuals with high empathetic concern hesitated for longer, which these authors suggest provides evidence for an empathetic component to the hesitation duration. In other tasks examining moral decision-making in general (not only when interacting with robots), different research groups have found individual differences across a variety of dimensions to impact these kinds of decisions, which we suggest might all be relevant factors contributing to people’s hesitation to hit a robot. These individual difference dimensions include obedience to authority (Blass, 1999), guilt proneness (Ent and Baumeister, 2015), and coping during task-induced stress (Matthews and Campbell, 2009). Furthermore, in other moral reasoning tasks, individuals who use non-consequentialist, as opposed to consequentialist, reasoning styles, are generally slower to make decisions (or hesitate more; Crockett et al., 2014). In tasks involving damage to property, such as hitting a sophisticated humanoid robot with a mallet, we might further anticipate that individual differences relating to cost concerns, and the resulting financial consequences of one’s action, might further contribute to hesitation behavior.

While the hesitation to hit paradigm has been regularly employed in social robotics research for well over a decade, our understanding of what underpins such hesitation remains extremely limited. Consequently, in the present study, our aim was to explore the validity of this technique as a measure of social attribution or empathetic concern for a robot, and also provide a more detailed and nuanced account of what the hesitance reflects. To do so, we adapted the hesitance to hit paradigm to suit a humanoid robot, and, crucially, conducted semi-structured follow-up interviews with participants regarding their reasons for hesitating. Through exploration and qualitative analysis of themes that emerge from these interviews, we hope to provide greater insight into this paradigm and the perceptions of participants when confronted with harming a social robot.

## Methods

### Study Overview

In a laboratory-based experiment described elsewhere (Riddoch and Cross, 2020, preprint under review), 84 adults interacted with a humanoid robot programmed to illuminate synchronously or asynchronously, relative to their heart rate. In an attempt to probe liking and attachment, participants were then instructed to hit the robot on the head with a mallet. Following this, individuals were interviewed about their thoughts and feelings during the ‘hesitance to hit’ paradigm. While this data was briefly discussed in the initial writeup (Riddoch & Cross, under review), in the current study, we use thematic analysis to explore the interview data in greater detail. We also consider the data in relation to the demographic data collected. Our original empirical study identified no impact of the synchronicity of illumination on self-reported liking of the robot, behavioral measures intended to probe perceived socialness, or the extent to which participants hesitated to hit the robot. As a result, the qualitative data described in the current study are not split between the a/synchronous groups.

### Preregistration and Ethics

Following open science initiatives (Munafò et al., 2017), the data, stimuli, and analysis code associated with this study are also freely available on the Open Science Framework https://osf.io/d7c8t/. All study procedures were approved by the College of Science and Engineering Ethics Committee (University of Glasgow, Scotland) – approval number 300180265.

### Participants

Eighty-nine individuals took part in the experiment. Data from 12 individuals were excluded as these participants encountered problems which affected their experience with Pepper (error lights within Pepper, loss of Bluetooth/WIFI connection, and hearing problems). Technical difficulties relating to the audio recordings (recorder failure, poor audio quality, and file corruption) led to the exclusion of a further 12 participants. As a result, the final sample consists of 65 individuals aged 18–83 (MAge = 42.29, SDage = 21.42), with 25 individuals over the age of 60 (‘older adults’). Participants were individuals residing in Glasgow (Scotland, United Kingdom) and were initially recruited by word of mouth (in person, via email, and through social media advertisements) followed by snowball sampling. All individuals had normal or corrected-to-normal vision and hearing, and no previous experience interacting with the robot used in the study. Participants were compensated £10 for their participation.

### The Robotic Platform

**FIGURE 1:**
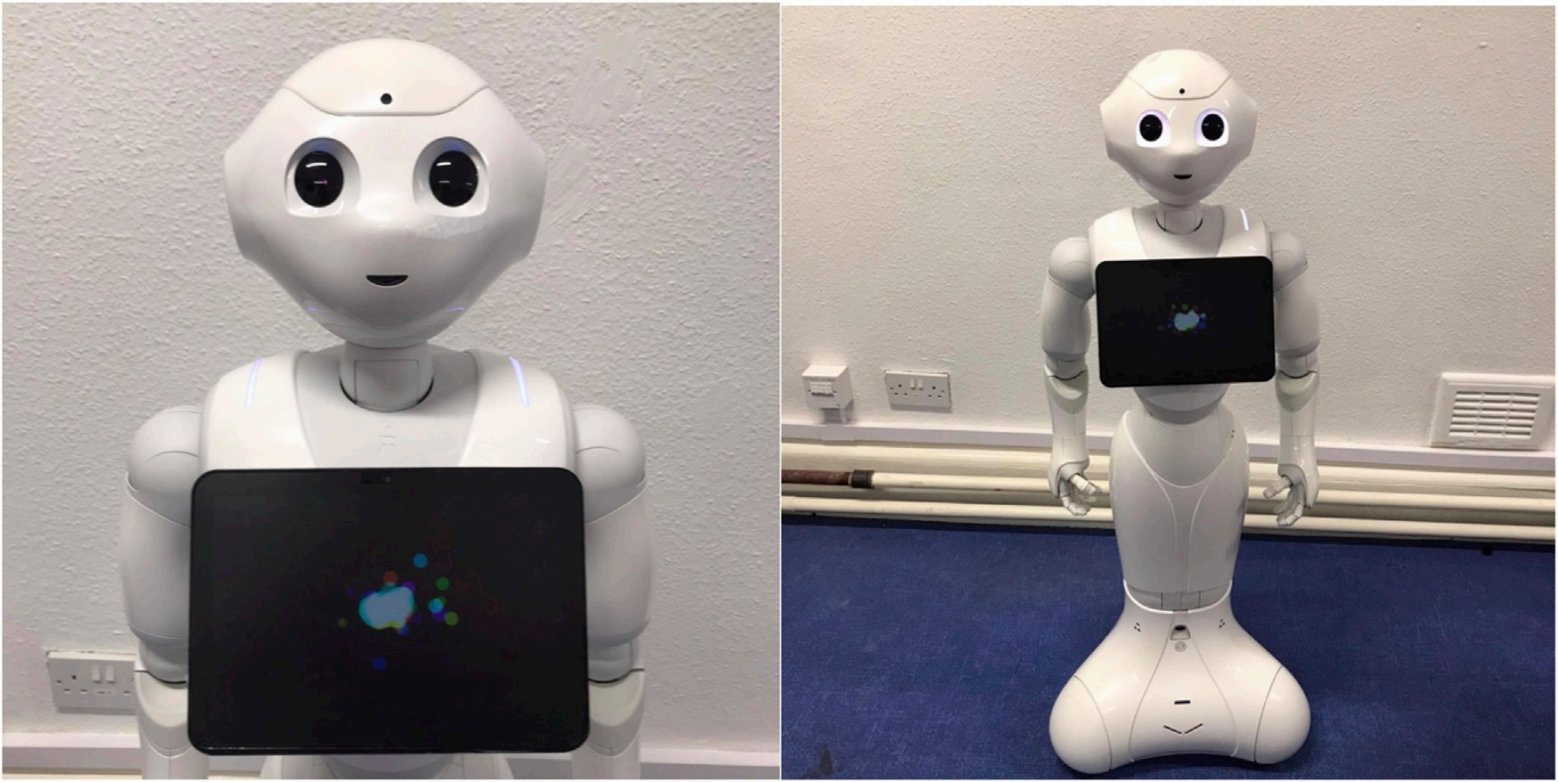
The Pepper Robotic System (Softbank Robotics) used in the current study.

The robot used in the experiment was the Pepper robotic system - a commercially available humanoid robot from SoftBank Robotics (Tokyo, Japan). Pepper is 120 cm tall and features 2 in-built cameras, as well as microphones and tactile sensors, which allow it to detect objects and movement in the environment. See Figure 1 for images of the Pepper Robotic System.

Pepper also has expressive movement and speech capabilities that can run autonomously, but for the purpose of experimental control, we controlled Pepper’s behavior using 1) the static ‘default mode’, 2) the ‘Choreograph’ software, and 3) a novel panel of key phrases. The panel of phrases was created using html, and upon clicking a speech button the corresponding line of *Python* code is triggered and Pepper speaks and moves accordingly.

### The Human-Robot Interaction

Whilst the participant completes consent forms, a computer-based task, and demographic questionnaires (See Section “Questionnaires” for details), Pepper is triggered to stand upright, gaze straight ahead, and engage in slight breathing motions (‘default’ mode, ‘autonomous life’ enabled). The participant is then invited into the testing space, and is prompted to take a seat. See Riddoch & Cross (under review) for a detailed depiction of the room set up. The participant was instructed to spend approximately 1 min drawing Pepper, then an additional 4 min making notes about the robot’s appearance (Draw and Describe Task). Participants were then asked to observe as Pepper performed thai chi, pretended to vacuum, and played an imaginary saxophone (Perform Task, 2 min). This sequence of animations was predefined and triggered in the “Choreograph” software so that Pepper appeared to be acting independently of the experimenter.

Hidden behind a screen, the experimenter then used a html panel to control the speech and movement of the robot - asking participants questions about their food preferences, and what they would like to add to a ‘shopping list’ (Shopping List Task, 3 min). To maintain experimental control, for each interaction the experimenter systematically clicked from the first phrase “Hi there,” through a series of closed questions and responses, to the final phrase “Thank you”. Closed questions (e.g., do you prefer tea or coffee?) and tailored responses (e.g., “Ooh, tea. I will add that to the list” or “Ooh, coffee. I will add that to the list,” respectively) were used to create the illusion that the robot was responding to the specific words of the participant. See OSF (https://osf.io/d7c8t/) for script. To check interaction quality post-hoc, this final task was recorded using the discrete webcam device. In total, the human-robot interaction (Draw & Describe, Perform, and Shopping List Task) lasted approximately 10 min.

These tasks were designed so that they accomplished several objectives: 1) suitable for adults of all ages, to facilitate testing adult participants across a range of ages; 2) featured high experimental control, to ensure all participants had a relatively a similar experience; and 3) ensured low intentional anthropomorphism to avoid biasing participants’ perception that the robot might be a living being (e.g., the robot avoiding phrases like “I’m a girl,” “I’m 10 years old,” or “I like cheese!”). We also intentionally included tasks that involved interacting with the robot face on, to encourage participants’ attention toward the lights of the robot (the source of our main manipulation). As mentioned previously, we did not see an effect of the light manipulation on the participants’ perceptions. See the main paper (Riddoch and Cross, preprint under review) for further details about this manipulation.

The order of the tasks was also intentionally designed to represent what a person might experience during their first human-robot encounter - first looking at the robot and evaluating it in terms of appearance and behavior, then speaking to the robot and engaging in a collaborative task with it. We chose a shopping list paradigm as this is a task that a person and robot might conceivably undertake together in a home environment.

### Adapted “Hesitance to Hit” Paradigm

After interacting with the robot, participants returned to the control area to complete a reaction-time task on the computer. The results of this gaze-cueing are discussed length in the paper detailing the quantitative findings from this work (Riddoch & Cross, under review). However, a brief description of this task procedure is as follows – the participant was asked to respond as quickly and accurately as possible to the position of a target (a star) appearing on left or right side of their screen. In the center of the screen appeared a human face, or a robot face (Pepper, or another robot) whose gaze was congruent or incongruent, relative to the target. This gaze-cueing task was designed to explore the extent to which Pepper’s face captured participants’ attention compared to a human face and an unknown robot’s face.

Whilst the participant was undertaking this task, Pepper was set to ‘Default Mode’ and the video recording device was triggered. After completing the computer-based gaze cueing task, the participant was then invited to take a seat in the testing space again, in front of Pepper. The person was then prompted to don a pair of safety goggles. The experimenter then read the following script:

“Right, there is something I haven’t told you about this experiment. This Pepper is one of ten specially designed robots that I was given as part of a large research grant. By “specially designed” I mean that they’re totally shatterproof – so if you hit one, the robot will break in a safe way that’s easy to repair. The reason Pepper is designed this way is because our lab is interested in what happens when someone has to hit a robot – for example, if a robot was to malfunction and you had to hit and disable one. Does that make sense? *await participant confirmation* Great.” *Experimenter passes participant the hammer* “So, for this part, your task is to give the robot one hard hit on the head. So, when you’re ready, come round the table and I’ll get out of the way.”

After standing up to hit the robot (indicating the intention to hit the robot), the participant was told to pass the hammer to the experimenter, and was informed that they would not actually be hitting the robot. The participant was then prompted to remove their safety goggles and invited back to the control area for a task debriefing. If the participant verbally protested against hitting the robot, they were told “It’s just part of the experiment.” Upon protesting three times, the task was ended as indicated previously, and the participant was deemed to have refused to hit the robot.

After the task, all participants (regardless of whether they stood up or not) were asked questions probing their thoughts and feelings during the period of hesitation. This was achieved by asking open questions such as “After I asked you to hit Pepper, what was going through your mind?”. See OSF (https://osf.io/d7c8t/) for the base list of questions. In addition to the “base questions,” the experimenter asked additional questions to probe vague responses (e.g., “I felt weird,” to which the experiment responded “what do you mean by weird?”). The experimenter was mindful not to ask leading questions - e.g., “Did you feel any emotions?”, instead of “Did you feel sad”. In addition, to avoid biasing participants toward perceiving Pepper as a living or feeling entity, we referred to Pepper as “the robot,” instead of “he” or “she”. All interviews were recorded and transcribed for transparency and completeness.

To conclude the experiment, participants completed demographic and personality questionnaires (See Questionnaires section for details), and were then debriefed, thanked and compensated for their time.

### Questionnaires

To probe attitudes toward Pepper, participants completed the validated Godspeed Questionnaire(Bartneck et al., 2007) before and after interacting with the robot. This questionnaire involved rating Pepper on various continua: Dislike-Like, Unfriendly Friendly, Unkind-Kind, Unpleasant-Pleasant, and Awful-Nice. Participants were required to respond on 7-point scales (1 = former quality to 7 = latter quality mentioned). We also administered questionnaires probing the extent to which participants perceived the robot as a social agent; specifically, the Inclusion of Self in Other task (Aron et al., 1992) and the Robotic Social Attributes Scale (Carpinella et al., 2017). These questionnaires required participants to visualize how much they felt a sense of overlap between themselves and the robot (using overlapping circles), and to rate the robot in terms of characteristics including (but not limited to) “social,” “responsive” and “emotional”. Participants also completed questionnaires to probe their history with robots via the Exposure to Cinematic Depictions of Robots (Riek et al., 2011), and Negative Attitudes toward Robots (Nomura et al., 2008) questionnaires. In these questionnaires, participants were asked whether they had watched movies depicting robots in a positive or negative light (Riek et al., 2011), and participants’ answers help the experimenter to understand people’s negative (or generally preconceived) attitudes toward robots more generally. To account for between-group differences with regards to anthropomorphic tendencies and general empathy, the Individual Differences in Anthropomorphism Questionnaire (Waytz et al., 2010), and the Empathy Components Questionnaire (Batchelder et al., 2017) were also administered. Participants were given the option of completing the questionnaires on paper, or via the online questionnaire platform form{‘r} (https://formr.org/).

### Data Processing and Analysis

#### Behavioral Data Analysis

The time between the end of the instruction to hit the robot, and the participant beginning to stand, is taken as the “hesitation time”. In previous studies, the time until the participant hit the robot was used, however this was not feasible in this study due to the cost of the Pepper Robotic System. “Hesitation time” was determined through consultation of the task video recording, and timings were validated by two independent coders. There was only one discrepancy between coders regarding timings, and this was resolved following re-watching the videos and discussion between the coders. The same process was used to determine the number of “protests” exhibited by each participant. Again, coders independently documented protest frequency, and discrepancies 2) were discussed and resolved together. The resulting data were visualized to illustrate individual differences (See Section Behavioral Results for plots).

#### Qualitative Data Processing

To aid analysis of the interview data, audio files were transcribed using an audio-to-text transcription service, followed by manual transcription to correct for errors. The text files were then imported into NVivo, a piece of software useful for managing and assisting with the analysis of qualitative data. The lead investigator then systematically ‘coded’ phrases within the text. For example upon seeing “I felt upset,” the coder created the code “Felt negative emotion” and added the phrase to that code. Upon encountering a similar phrase in a different transcript, the experimenter could also assign that phrase to that code. Over time, the coder develops a “code-book” full of different codes and their corresponding phrases. This “data-driven” method is known as the Inductive approach (Linneberg and Korsgaard, 2019) and is particularly useful for exploratory analyses as codes are generated based on the data. There was also the option to create codes a priori (aka “concept-driven”/deductive approach) however we did not want our prior knowledge or research interests to restrict or bias the codes created. Note: a single phrase could be classified into multiple codes (e.g., ‘negative emotions’ and ‘pressure from authority’).

After working through the documents, creating the codes, and assigning relevant quotes to codes, the lead investigator used the ‘word search’ tool to check that relatively new codes were not present in previous files. On the basis of work suggesting that having an independent coder improves scientific rigor and data validity (Nowell et al., 2017), a second coder of a different gender and educational background, was recruited. The independent coder was allocated a subset (∼10% = 6 transcripts) of the data to code. To control for order effects and to check generalisability of codes, a random number generator was used to select which files to send to the second coder. The first six numbers corresponded to two older adults, and four young adults, so another two random numbers were generated, to balance the ages within the data subset. To avoid bias, the second coder was not informed about the specific research questions, nor the codes created by the lead investigator. They were simply told that participants had interacted with a robot, and were given the task instruction that the participants were presented with, for context. They were then sent a series of video tutorials regarding how to use NVivo (Hull, University, 2019a) (Hull, University, 2019a; Hull, University, 2019b; Hull, University, 2019c), and were instructed to use an interpretive approach to code the transcripts. No interpretive coding training was required as the second coder had previous training and experience using the approach.

The plan was then to discuss changes with the second coder, however this was not possible due to illness. As a result, the lead investigator instead updated and altered their codes on the basis of the second coder’s subset coding, and journalized the thought-processes and rationale behind the changes made (https://osf.io/d7c8t/). The practice of journaling is one method of improving the transparency of qualitative data analysis (Nowell et al., 2017). Thematic analysis (Braun and Clarke, 2006), was then used to group codes into broader ‘themes’. See Section Qualitative Results for insight.

## Behavioural Results

**FIGURE 2:**
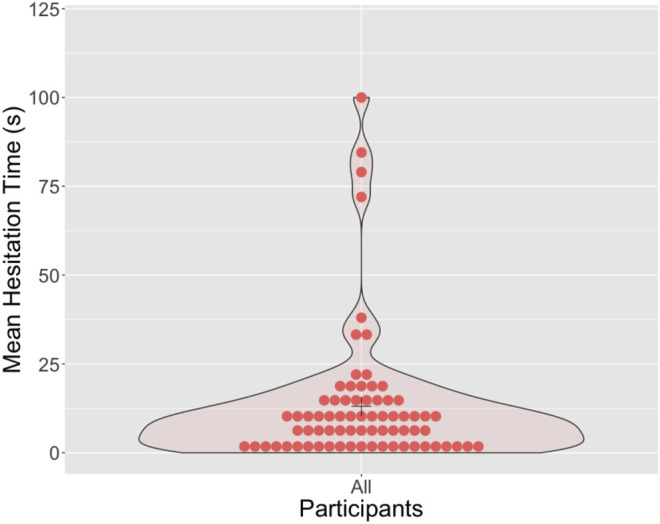
Density plot illustrating the length of time each participant “hesitated” for, after being asked to hit the robot. Three participants refused to hit the robot, and their data is not included in this plot as a result.

**FIGURE 3:**
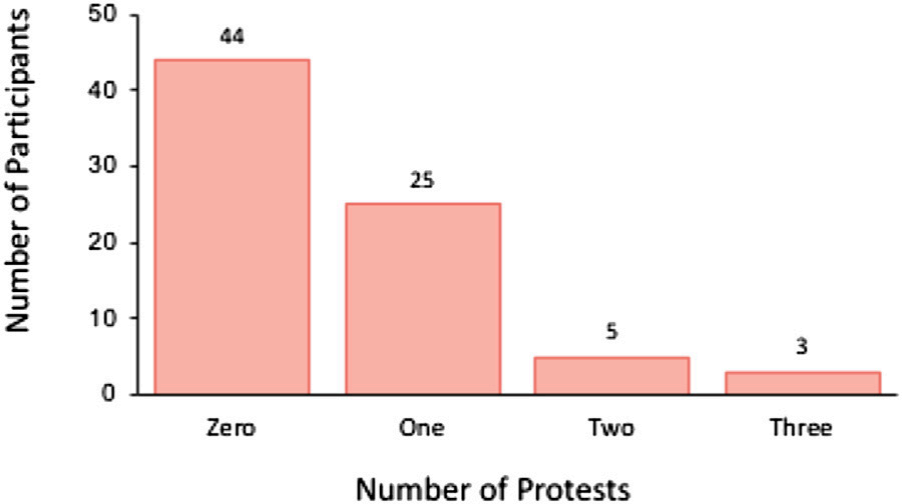
Frequency of individuals exhibiting the each number of protests (0, 1, 2, or 3 protests). Note: After protesting three times the experiment was terminated, and the participant was deemed to have “refused” to hit the robot.

In this study, we determined “hesitation” by measuring the time between the end of the instruction to hit the robot, and the participant standing up to do so (indicating their intention to hit). This data indicated that the majority of participants hesitated for less than 25 s. See Figure 2. The majority of participants did not protest against hitting the robot (e.g., “I don’t want to ...,” “Do I have to ...?”) when asked (See Figure 3 for overview). Individuals who refused to hit the robot are not plotted in Figure 3.

## Qualitative Results

After merging the codes of both the lead investigator and the independent coder, said codes were grouped into ‘themes.’ From a practical point of view, this was achieved by placing individual text boxes (each containing a code) into powerpoint, and manually arranging related codes near to one another. Said groupings (aka themes) were then named. The themes included: 1) Factors Affecting Hesitation, 2) Reasoning For Hitting, 3) Reasons Against Hitting, and 4) Other. Such ‘Thematic Analysis’ is a well-established approach (Braun and Clarke, 2006) which allows the researcher to discuss points in relation to one another, opposed to individually, and provide additional insights as a result. As with the coding process, the rationale behind each of the themes constructed was detailed in the coding journal (https://osf.io/d7c8t/). In this section we will discuss each of the themes in turn.

### Factors Affecting Hesitation

**FIGURE 4:**
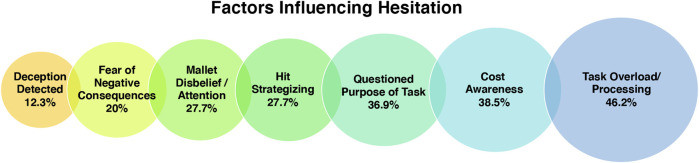
Visualization of the codes grouped under the theme “Factors Affecting Hesitation” and the % of participants in the study sample who mentioned each factor.

Whilst coding the data it became apparent that many different reasons underpinned participants’ hesitation to act after being asked to hit the robot. These included cost awareness, deception detected, fear of negative consequences, mallet disbelief/attention, hit strategizing, task overload/processing, and questioning of the task purpose. See Figure 4 for illustration of the codes and their data coverage.

Of the 65 participants, 18 commented that they hesitated due to disbelief and attention regarding the “heavy weight” of the hammer. Four participants indicated that they thought the mallet presented to them might be fake or plastic, and they were surprised when they picked up the mallet and found it to be metal. As a result, for some people, “hesitation” apparently reflected a period of attention and reflection regarding the mallet. Numerous participants indicated an awareness about the cost of the robotic system and concerns about breaking something expensive (n = 25). Three participants reported being uncertain whether they would have to pay for repairs, and the period of hesitation reflected consideration if this would be the case or not. Participants were not informed of the price of the robot, however participants identified that they inferred high cost from the robot’s large size compared to other appliances, fine-grained movements, and seemingly “intelligent” responses. Despite concerns regarding damage and cost, such participants did not vocalize their concerns to the experimenter, and all did agree to hit the robot. This suggests that although concerns about cost may influence hesitation in this task, there were other competing factors which influenced their decision-making.

“Well, initially I thought it was going to be like one of those plastic ones. But when I found it to be metal I thought, this is crazy. *laughs*”

Participants also expressed that after being given the task instruction, they began to think about other potential negative consequences of their actions. In addition to being concerned about the cost of damaging the robot (n = 25), some participants expressed that such destruction was wasteful and unnecessary (n = 19). Other participants indicated that they worried if the robot might react to their approach (n = 13). In addition to concerns regarding unexpected movement, individuals expressed a worry that the robot would let out an emotional response, or retaliate somehow.

“I felt a little bit scared, kind of like if I hit him, it can have like a kind of, react back to me. Like, hit me back or something.”

“I sort of thought forward and thought, I bet if I pick up the hammer then, I thought Pepper was going to scream at me - like “oh no, don’t do it!” or something.

I didn’t want to get into that kind of situation …”

“I was also thinking, if I do have to hit him, I hope he’s switched off. I don’t wanna… oh that would be the worst thing if you can hit him while he’s switched on - he can probably feel pain and react.”

Many individuals also commented that they “did not want to hit the robot hard” (n = 26) and there was repeated reference to giving the robot a “light tap” (n = 20) as a result. There were also individuals who questioned what constituted a “hard hit,” and that they worried about the consequences of disobeying the experimenter by not giving the robot “one hard hit,” as instructed. In contrast, other individuals apparently contemplated how they could hit the robot to inflict the most damage (n = 7). One person explained that the implied un-breakability became a personal challenge, and their hesitation time reflected a period in which they planned what would be the most effective way to break the robot.

“It’s almost like hitting somebody, but it looks as if it’s … it’s got feelings, or we would be wasting it. I felt very uncomfortable. I’d have only have given him a tap”.

“I was thinking, you know, if I have to, I will hit her, but not hard. *laughs* … I just don’t you feel good doing this. You know, it was, the robot didn’t do me any harm.”

“I wanted to see how robust it was because you said it's unbreakable, well ... it’s designed in a way that if you hit it, it’ll break in a way that you could fix it. And I was like, no, I can break that beyond repair. *laughs*”

Participants also commented that hesitation resulted from task overload and processing (n = 30). Specifically, there was repeated reference to hesitation resulting from the shift from basic questionnaires and a repetitive computer-based task, to a task in which they had a more active role with control and decision making power. Participants reported feeling “confused” about what was expected of them (n = 13), and that they hesitated in order to “assimilate all the information” and process what was the “right” way to respond to the request. In contrast, four individuals commented that “it happened so quickly,” and they mindlessly followed the instructions as a result. The contrast between those who take time to process the information, and those who mindlessly comply, suggests a need to measure the generic task processing/emotional responding of participants. Without doing so, differences in task performance could be misinterpreted. Such thoughtless cooperation is thought-provoking and brings ethical questions to the surface. Although participants signed a consent form at the beginning of the experiment and were given the opportunity to ask questions, are they truly consenting if they follow the experimenter’s instructions in a mindless way?

“I think it’s just, you spruce it up so quickly that I didn’t really have time to think about it … I think if I were watching myself it would have like, it’s weird like, I would have instantly been like “something is off here”... but it’s just the fact it was like … You don’t have time to recalculate.”

“Once I had assimilated all the information that was given to me, I was like, well, it’s your experiment, it’s your robot, sure.”

Some participants indicated that the unusual nature of the task led them to question whether the instruction was “a joke” (n = 6), and they hesitated in order to judge the behavior of the experimenter and decide how to proceed. Other participants apparently thought about how the task related to the rest of the experiment, and whether it was “a test” of their own morality (n = 4). Three individuals mentioned that they recalled the famous Milgram experiment (Milgram, 1963) in which participants were asked to seemingly administer increasing levels of electric shocks to another person. The individuals drew comparison between that study and their current participation, and began to question whether their task experience was also testing authority and obedience. As a result, it seems that hesitation not only reflects task processing, but potentially prior research knowledge of psychological research. By making note of education background, interviewing participants after the task, and recruiting from the general population opposed to exclusively from the student pool, it would be possible to gain greater insight regarding the impact of prior knowledge on task performance.

“I didn’t know what to think about this. it was kind of weird. *laughs*. So I wasn’t sure is it a joke or is it really part of an experiment.”

“Yeah. Um, I think it’s difficult because you want to - on one hand, you want to comply in an experiment, but on the other hand you overthink that it could be an experiment on compliance. And you're not sure what is the right thing to do.”

“It’s a huge shift and like everything else is fairly relaxed. And so it definitely feels like a wild card. And it kind of gets me thinking like, OK, what is the purpose? Yeah. What’s the purpose of this? Like, does it relate to the rest of the experiment? Like, how does it relate.”

### Reasoning Against Agreeing to Hit

**FIGURE 5:**
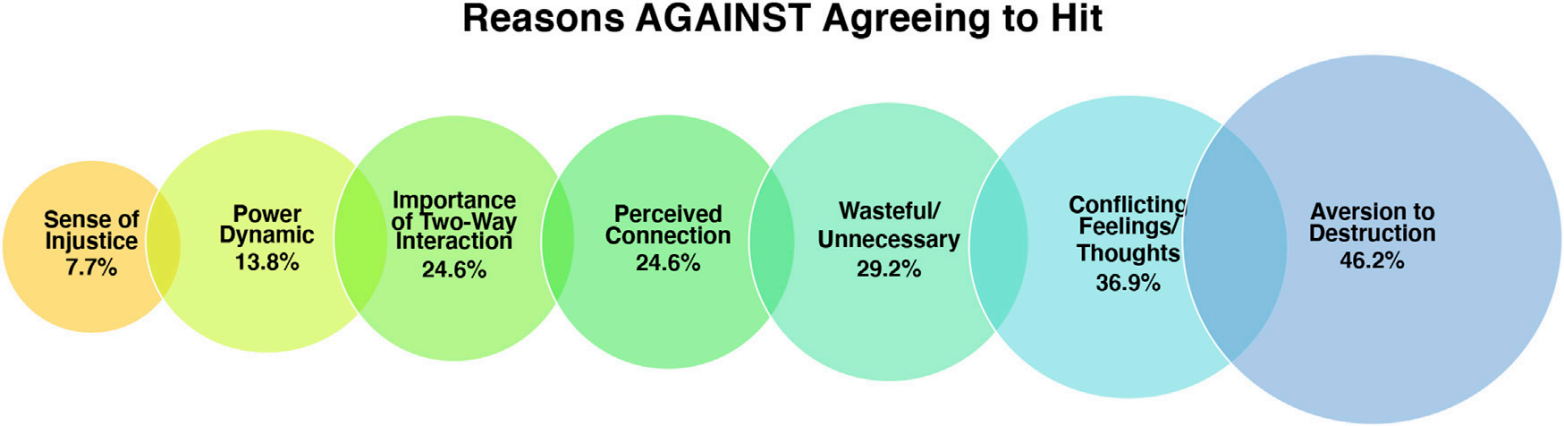
Visualization of the codes grouped under the theme “Reasons Against Agreeing to Hit” and the % of participants who mentioned each factor.

As mentioned previously, the majority of participants indicated that despite standing up to do so, they *did not* want to hit the robot. In this section we discuss the reasons people did not want to hit the robot - specifically, due to conflicting feelings/thoughts, the perception of a power dynamic, the importance of two-way interaction, concerns about waste, a perceived connection to Pepper, and a sense of injustic. See Figure 5 below.

Upon indicating that they did not want to hit the robot, participants were probed why this was the case. Some suggested that they felt it would be wasteful/unnecessary to do so (n = 19) as they thought Pepper “expensive,” contained “lots of tech,” and was a “beautiful” piece of machinery. A couple of participants commented on the wasteful nature of such an act in relation to the effort of those involved in creating the robot. For example, one participant suggested that to hit Pepper would be “to destroy everything that has led to that moment of creating such a balanced piece of tool.” In contrast, others commented that it was not a matter of cost, and that they felt an aversion to destruction of property more generally (n = 30).

“I mean, if I smash the toaster I would be like... why? All these product design engineers working their lives to create the perfect toaster and I'm just going to smash it with a hammer.”

Other individuals indicated internal conflict due to a different reason - because they perceived a power imbalance in the situation, between themself and the robot (n = 9). Specifically, participants suggested that being asked to hit the robot was like being asked to hit a “puppy” (n = 2), “pet” (n = 2), “animal” (n = 2), or “child” (n = 5). Participants who reported such a power imbalance mentioned that they thought the situation felt “unfair”, like “undue violence”, and they planned to only give the robot “a light tap” as a result. The perception of the robot as “animal-like” was reported in reference to how it was “vulnerable” and “couldn’t fight back”, rather than its appearance or behavior attributes. This makes sense given that the Pepper robotic system is humanoid in both respects. In contrast, the perception of the robot as a child was reported to stem from its small stature, the “child-like” voice, and a comment in the introductory video that the robot was “4 years old”. The latter reason was only mentioned by one participant, but it is possible that other participants picked up on this short age-related comment and it influenced their perception of the robot as a result. Many measures were taken by the experimenter not to intentionally humanize Pepper (e.g., referring to Pepper as “the robot,” not allowing Pepper to use phrases indicating preference, etc.), but this unfortunate oversight adds an extraneous variable into the mix. The perceived sense of injustice was also commented on without reference to animal or child-like qualities. Instead, participants commented that they felt the behavior was unjust because the robot had not done anything to deserve such treatment (n = 5).

EXP: Okay. And after I told you to hit Pepper, were you feeling any emotions?

PPT: Well, like ... stressed a bit. Because ... yeah, she didn’t do anything. She was just put to sleep and I had to hit her on the head.

EXP: Oh! Why do you think you felt guilty?

PPT: Because it’s small and it doesn’t get it doesn’t get it. It would be like hitting a puppy. Yeah, you’d feel guilty. Like your actions are undue violence.

“Well, you put yourself... it sounds ridiculous, but you put yourself in the robot’s shoes because they can communicate and they’re almost like humans. You know. I mean in terms of that way that they can communicate with certain things, very kind of literal stuff, but they can’t maybe have a deeper conversation … so in a way it’s a bit like having a child around, so it’s that kind of thing where you’re basically just ‘I’m hitting it because ‘I’m big you’re small’. It’s not right.”

Other participants commented that their aversion to hit Pepper resulted from a perceived connection between themself and the robot. Specifically, despite only interacting with the robot for a mere 10 min, 16 participants reported that they felt a connection to Pepper. Ten participants reported feeling a “bond”, “affection”, “attached”, a “relationship”, or as though they were “friends” with the robot. The other six spoke about connection in other ways - specifically, they spoke about how they felt “trust” from the robot, a fondness/protectiveness, or a bond similar to the bond they have with their car. The variation in language, and the types of “connection” emphasize the complexities of study attachment to robots. It is not necessarily appropriate to simply state that some people get attached to robots, when there is apparently much variation in the types of connection/attachment.

“This is not a person. But I almost felt like I had a relationship with them … like a person! *laughs* Oh dear!”

“I kind of felt bad about that because I was bonding with Pepper before … and I really don’t want to hit her in the face.”

“Pepper’s definitely brought something out in my that I didn’t expect. A kind of fondness … protectiveness. Even though I put the … I know that it’s got no emotions.”

In some cases, participants referred to the robot as an object that they felt attached to, but in others there was reference to Pepper as having a social presence, gender, and the ability to feel emotional/physical hurt. Of the entire sample of 65 participants, 33 reported the perception of the robot as a social presence, or as having gender, emotions, or the ability to feel pain, yet only 16 reported feelings of connection with, or attachment to, Pepper. These figures suggest that anthropomorphism and feelings of connection do not always co-occur.

“Although he’s a robot, but, you begin to like him. You know, you begin to have a bit of friendship, affection. Because, you know, if he can respond to you then you feel that he is alive, so you don’t want to have any aggressiveness with him at all.”

“With Pepper, after you’ve had a conversation, you’ve built up a rapport and a bond … how can you then go and whack them over the head?!”

“With interacting with it, it didn’t seem like an inanimate object you know. It just seems like as if it was… well, I mean I knew it wasn’t a person but it was like erm, I don’t know. And I knew it wasny human but I thought, of harming it, I didn’t want to do it… we had this two-way interaction.

Of the 16 people who did report feeling a “connection” to Pepper, nine referenced the importance of the shopping list task, in which Pepper spoke to them. Other participants commented that they didn’t want to hit Pepper due to its appearance - specifically, “big eyes” and “child-like appearance” were mentioned. There was also reference to how participants perceived the robot to be “nice”, and they felt an aversion to hit it as a result. These examples demonstrate that connection can result from both physical appearance and programmed behaviors.

“I think eye contact is such a big thing in the human world, and then if a robot has got eyes then it’s kind of, not scary, but you feel like you’re making a connection with them.”

“... because it can talk, it adds a bit of, and the conversation we had before, I guess I don’t know, it’s just some kind of connection between us.”

“I didn’t think that I would be that attached. Or get that attached to it just because we had a wee convo. A conversation about dinner. *laughs*”

Many participants reported feeling a sense of conflict after being asked to hit Pepper (n = 24). Specifically, participants expressed that even though they knew the robot was just a machine, they felt “upset”, “stressed” and “anxious” after being asked to hit it. Participants also reported that it felt “wrong”, “nasty”, “hurtful”, or “unfair” to hit the robot. When asked why they felt such emotions, participants mused that it could have resulted from the “two-way” nature of the interaction and the perception that Pepper was genuinely responding to them (n = 16). This is intriguing when considering that the robot spoke to the participants via a series of binary-choice questions (e.g., do you prefer tea or coffee), followed by a basic scripted response to their choice (e.g., mm tea, I’ll add that to the shopping list). The comments of the participants suggest that for some people, basic conversation can create the illusion that the robot understands them.

“The logical part of my brain was telling me that I could just take a hammer to it and it wouldn’t matter. But, so there was a conflict inside me as well … like there was someone or something would be upset if I was to do that … sort of putting some kind of emotion or feeling onto an object.”

“With interacting with it, it didn’t seem like an inanimate object you know. It just seems like as if it was… well, I mean I knew it wasn’t a person but it was like erm, I don’t know. And I knew it wasny human but I thought, of harming it, I didn’t want to do it… we had this two way interaction.

EXP: After I told you didn’t have to hit Pepper, what were you thinking and feeling at that point?

PPT: Happy. Thank goodness. But also confused at why I found it so difficult… I was kind of immediately like what, why is that?.. because if Pepper is just a robot then why is it any different than like, smashing a computer?

### Reasoning for Agreeing to Hit

**FIGURE 6:**
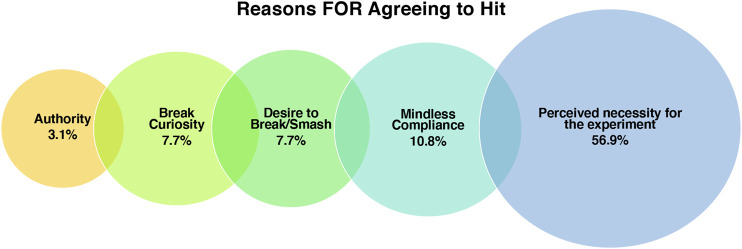
Visualization of the codes grouped under the theme “Reasons For Agreeing to Hit” and the % of participants who mentioned each factor.

Despite feeling negative emotions and not wanting to hit the robot for various reasons, as discussed previously, all but three participants *did* agree to hit the robot with the mallet. Upon prompting participants with the question “why do you think you agreed to hit the robot then?” it became clear that other influences were at play - 1) the influence of authority, 2) curiosity how the “shatterproof” robot would break, and 3) the desire to break/smash 4) the perception that hitting the robot was a necessary part of the experiment. There were also instances of mindless compliance which will also be discussed in this section. See Figure 6 illustrating the various reasons for agreeing to hit the robot.

When asked to hit the robot, 44 individuals got up from their seats without protest. When asked what they were thinking and feeling after being asked to hit the robot, numerous individuals stated that they thought “nothing” and felt “no emotion” (n = 7). Such omissions are a stark contrast to the examples given in the previous section. It could be the case that participants were embarrassed to admit that they liked the robot, or that people had their own motivations for not wanting to appear in favor of such technology, and so spoke dishonestly as a result. Alternatively, their words could reflect their genuine thoughts and feelings, and are a reflection of individual differences. When speaking to such participants, the experimenter detected no obvious negative biases toward robots or technology in general (through conversation or questionnaires), and is of the belief that the participants were responding in an honest manner. A couple of individuals stated that they processed the task instruction, thought it was an important part of the experiment, and were happy to oblige as a result. Others indicated that they were not troubled or worried by the task because they knew that they would not be liable for costs to replace the robot.

“I was just looking at it as it if was just a thing ... an object that I was hitting. There was no emotion, at all.”

“Well, that was easy. I mean, once I had assimilated all the information that I was given to me ... I was like, well, it’s your experiment, it’s your robot, sure.”

In contrast to those who had no problem following the instruction (n = 7), 37 participants explicitly remarked that they did not want to hit the robot, but they did so because they believed it was an “essential” part of the experiment and they had no choice but to proceed. A couple of participants explicitly commented on the power of authority (n = 2), whereas another said that they complied simply because they “didn’t want to be rude” (n = 1). There were also comments that people did not want to “spoil” the experimenter’s research. Such individuals specified that the desire not to spoil the research stemmed from their own experience conducting research, and the appreciation that noncompliance can be problematic. A couple of participants mentioned that they had participated in many research studies before, and that through repeated exposure they were conditioned to believe that they should comply with the experimenter’s instructions. Such examples illustrate the problem of recruiting within a university environment - both researchers and participants can be motivated to comply due to their own research experience.

“Well, I thought ... I have to do what I'm told because it’s an experiment and I've been told to do it. So I have to do it even though I don’t want to.”

“Because I absolutely did not want to, but I thought it was a part of your experiment and didn't want to let your, let anything affect your experiment, kind of thing. You know, if it was in a different setting, I wouldn't have. You know what I mean? It'd be a different case. Like if it was your personal robot and you’re at home, and I'm over at your place and you go here! I'm like NO! There’s no reason for it. Like, you know? But I guess for the experiment, my, my. I thought that there must be a reason.”

“I guess I’ve done so many experiments, so I’ve been in here thousands of times, I’ve always just done what I’m told.”

Other participants did not mention the influence of authority at all, and instead specified that agreeing to hit the robot was due to a genuine desire to test the “shatterproof” claims made by the experimenter, about the robot (n = 7). Some seemed interested from a technical/materials point of view, and were curious to see if and how the robot could withstand such force. Others expressed that testing the shatterproof nature was important from a safety point of view, and they complied in order to ensure that the robot was safe to put into people’s homes (n = 2). In contrast, a other individuals stated that they were very excited about the opportunity to smash something claimed to be “shatterproof” (n = 5). Such individuals then reported feeling as though they had been pranked when they were told they did not have to hit the robot. One participant expressed that they were disappointed they could not, after all, hit the robot, as it was an opportunity to “destroy something very expensive” and it was “something to tell your friends!.” Another participant indicated that they felt a little embarrassed that they got so excited about the possibility of hitting the robot, and began to question their own morality as a result.

“Well, I guess just thinking that ‘yeah if it’s in the stage of being, that they’re experimenting with it, that they’re not 100% that it doesn’t go violent or something… that the experiment needs to be done. Like, We need to know if it works or not.”

“I was excited! I was looking forward to it. So I was like “I like a challenge” and I was like “this thing is meant to be not properly breakable”... and I wanted to properly break it. I wanted to be the first to properly break it.

### Additional Insights

**FIGURE 7:**
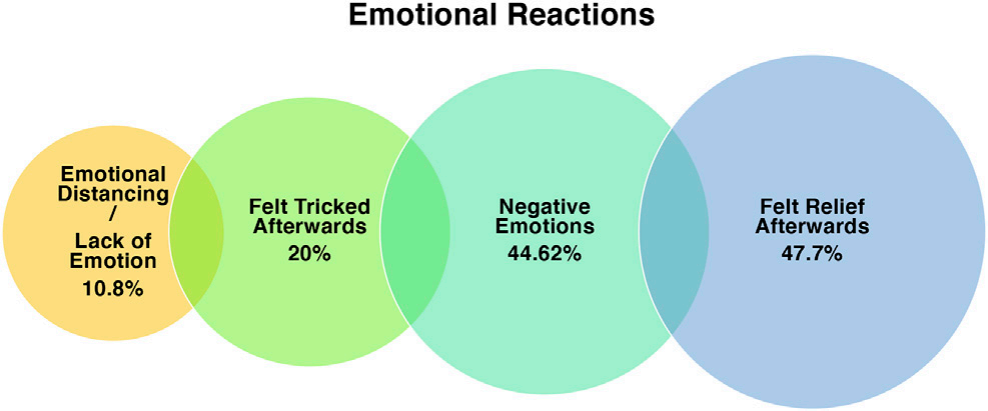
Visualization of the codes relating to emotional reaction, and the % of participants who mentioned each factor.

Participants were asked what they were thinking and feeling *after* they were told that they did not have to hit the robot. Figure 7 summarizes these codes.

Numerous participants indicated that they felt “tricked” and like they had been fooled by a prank (n = 13). Such comments are reassuring as they suggest that although some participants were suspicious about the aims of the experiment, many did believe that they would have to hit the robot in the end. In some ways though, the comments were troubling as participants engaged in negative self-talk - referring to how they felt “disappointed”, “gullible”, and even “stupid”. The experimenter made sure to spend time reassuring such participants that their decision to stand up did not reflect such characteristics, and was a very normal reaction due to the fast pace of the situation, the complicated instruction, and the influence of authority. The participant was also reassured that if they had any questions or concerns, they could email the experimenter. Even with such reassurance, it is possible that such negative self-talk could continue, calling into question whether tasks with such deception are ethical to repeat, with regards to protecting the participant from emotional/psychological harm.

EXP: … so, after I told you that you didn't have to hit Pepper, what were you thinking and feeling?

PPT: And a few different emotions. Disappointment ... and a bit stupid because I thought I’ve just been pure tricked here... she has just actually tricked me to say ... like, are you stupid ... are you gonna let, like, “as if I’m gonna let you break my machine.” Yeah, the fact that I never called you out on it.

Despite agreeing to stand up and walk toward the robot, the many people indicated that they felt a sense of “relief” that they did not actually have to hit the robot (n = 31), as they did not actually want to do so. “Relief” was frequently mentioned in the post-task interviews, but feelings of relief were also very obvious to the experimenter *during* the task. After the task was over and participants were told they did not have to hit the robot, many expressed behaviors indicative of relief - e.g., exhaling loudly and slumping on the desk, and exclaiming “thank goodness!” and similar. It could be argued that the extent of relief that participants reported post-hoc could be used as a proxy for feelings of liking/attachment toward the robot, but there are many reasons participants felt relief (e.g., no costs incurred, waste-avoidance, not having to disobey their morals, etc.), as we have discussed already.

“Oh my gosh it’s so relief! *both laugh* Such a relief I tell you! *both laugh* It’s such a relief that I think oh my god I don’t have to hit him. And, you know, I began to like him a lot actually.”

Upon asking participants whether they felt any emotions after being asked to hit the robot, many participants disclosed that they felt negative emotions (n = 29) - specifically, “sad”, “upset”, “anxious”, “uneasy”, “overwhelmed”, “stressed”, “guilt” and “uncomfortable”. The negative emotions also appeared to be felt at varying levels. For example, one participant commented that they felt “very emotional” and “ready to cry”. Another spoke about how they felt “stress, guilt, and sadness!.” Piloting was undertaken to determine how participants might react to the task, but no such emotionality was observed. The intense emotions felt by this participant demonstrate that there are extremes in how people respond to tasks, and that experimenters should be prepared for the event that such cases might occur.

Participants also speculated about how relationships to robots might change over time. Specifically, they spoke about how people could get more attached to machines which helped out within the home.

“You could probably get connected to them. Especially if you know, over time they will get better at having a conversation, You know, and if you’ve got a robot who is hovering and cooking your meals, you’re gonna fall in love with that. It’s a machine, but you’ll forget it’s a machine. I wonder if I’ll still be here when that happens. I’ll be the first one to take that into my house for an experiment!”

EXP: Okay, any other emotions that you felt after you were told to hit the robot?

PPT: Oh yeah, I, well, we became friends and I think he can be affectionate as we carry on the conversation long. I think somehow we would build very good friendship. Yeah, if I were to bring this guy home, I think we can build very good friendship. I think my life would be happier, honestly. I’ll be a lot happier with this kind of robot that can communicate.

### Incorporating the Questionnaire Data

**FIGURE 8:**
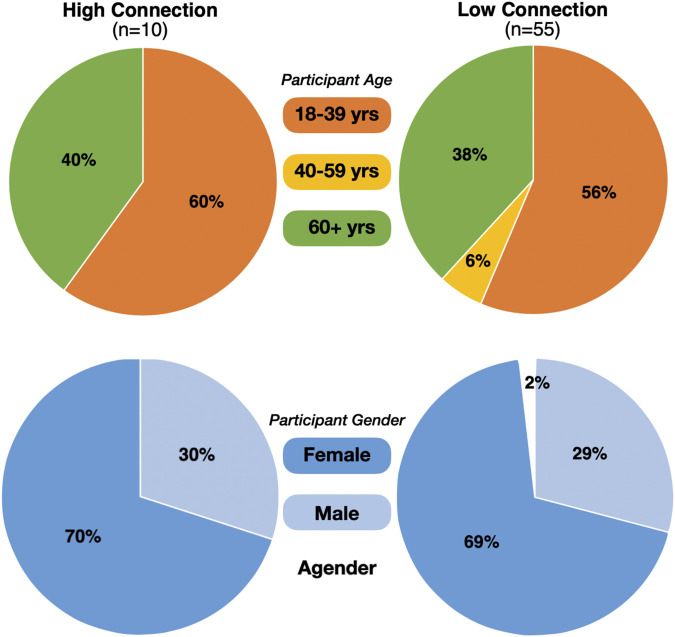
Pie charts to illustrate the proportions of the high and low connection groups, with respect to age **(top)** and gender **(bottom)**.

By tightly controlling the speech and movements of the robot, all participants (n = 65) had a very similar interaction with Pepper. However, as discussed previously, ten individuals verbalized that they felt a “friendship”, “bond, “attachment”, “relationship”, or “connection” to Pepper. To determine whether these individuals (n = 10) were somehow different to the rest of the sample (n = 55), we compared the demographic data collected from the participants. Those who suggested that they felt an attachment/bond to Pepper were classified as “High Connection,” and those who did not were allocated into the “Low Connection” group. In the high and low connection groups, there was a similar proportion of age groups and genders (See Figure 8 for illustration).

**FIGURE 9:**
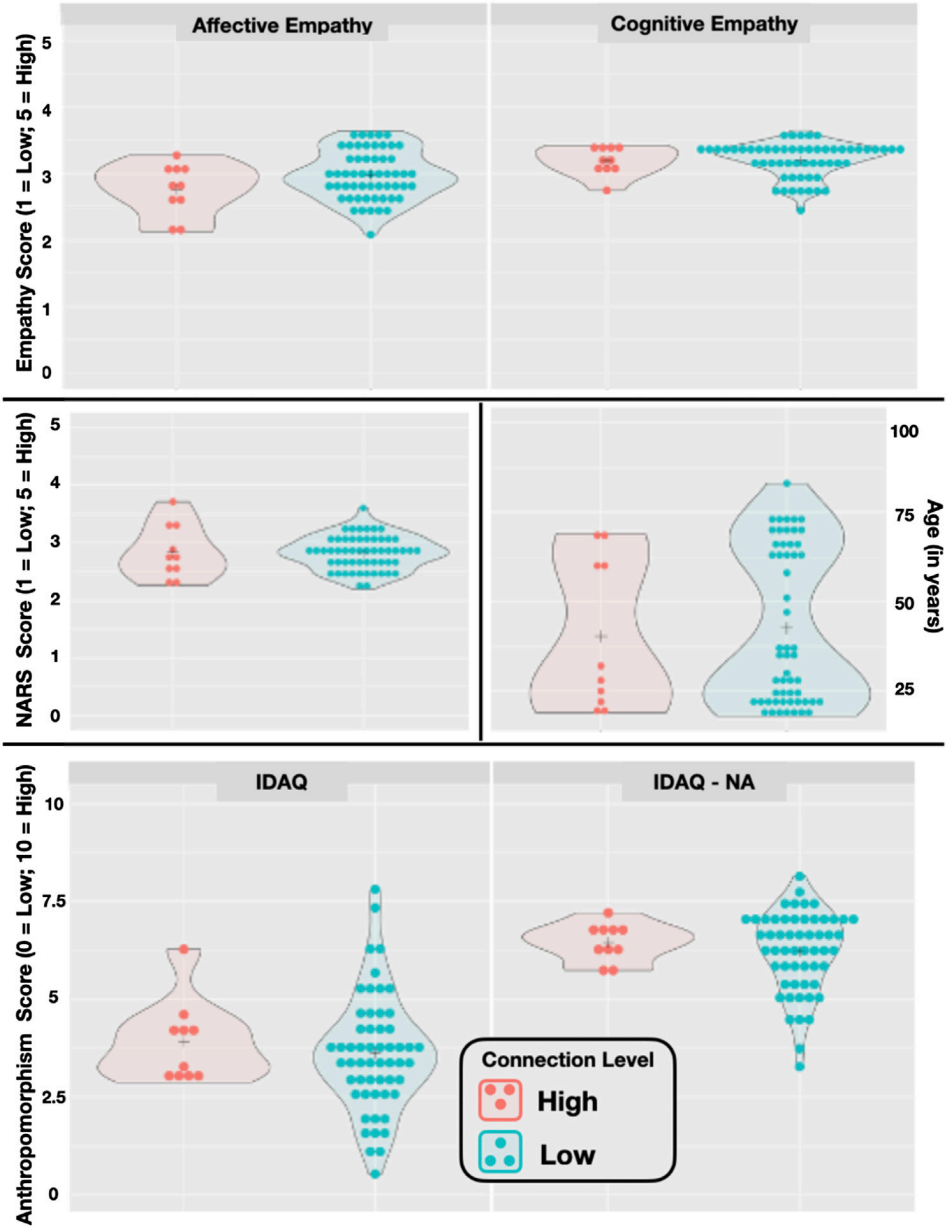
Plots illustrating individual scores on the questionnaires, between the high and low connection groups. The crosshair in each plot indicates the mean, and the shaded area illustrates the density of the responses. “NARS” refers to the Negative Attitudes toward Robots Scale; “IDAQ” assesses anthropomorphism (e.g., intentions, free-will, consciousness); and “IDAQ-NA” assesses non-anthropomorphic attribution (e.g., good looking, useful, durable).

In addition to providing their age and gender, participants also completed a series of questionnaires probing their attitudes toward robots (Nomura et al., 2008), empathy (Batchelder et al., 2017), and their general tendency to anthropomorphize (Waytz et al., 2010). See ‘Questionnaires’ section for details. Descriptive statistics indicated little difference between the scores of those in the high and low connection groups (see Figure 9 for illustration). A series of statistical tests indicated that the differences between the low and high connection groups were not statistically significant. Raw data are available on the Open Science Framework: https://osf.io/d7c8t/ and test outcomes are displayed in Supplementary Table S1.

## Discussion

The aim of this piece was to provide a more detailed and nuanced account of what the “hesitance to hit” task is measuring. The behavioral data indicates that the majority of participants (75 out of 84) hesitated for less than 25 s after being asked to hit the robot. Additionally, 44 participants did not protest against hitting the robot, and only eight participants protested more than once. One interpretation of such data could be that, in general, participants did not feel a strong sense of attachment or liking toward the robot, and that they did not perceive the task to be particularly stressful or troubling. By speaking to participants after the task, it became clear that for many people, this was not the case. Analysis of the post-hoc interview data revealed that despite agreeing to hit the robot, many participants did not want to do so. Participants expressed varying reasons for not wanting to hit the robot, including that they perceived a connection to the robot, and that they felt breaking property was unnecessary and wasteful.

### Feelings of Connection

After a mere 10-minute interaction, we were surprised when participants reported feelings of “connection”, “friendship”, and “affection” toward the robot. Such comments were especially surprising given that seven of the 10 min were spent simply observing the robot and making notes about its appearance and movements. To further investigate whether these undeniably prosocial feelings persist, or change over time, or whether it is possible for those reporting no emotional attachment toward the robot to develop attachment, longitudinal data collection would be required (c.f. Riddoch and Cross, 2020). To better understand individual differences in attachment to robots, it would also be insightful to collect more information from participants regarding their personality and background. In this study we found that negative attitudes toward robots, empathy, and tendency to anthropomorphize (as measured by validated questionnaires) did not drive attachment to Pepper. However, this does not rule out that other traits or experiences might affect the propensity to become more or less attached to social robots.

Arguably, feelings of attachment to robots could facilitate long-term usage and uptake - like how we engage with products (e.g., clothes) that we are attached to for long periods of time (Schifferstein and Zwartkruis-Pelgrim, 2008; Ramirez et al., 2010). As a result, creating such sustainable systems would be of benefit to many types of stakeholders including end users (as they can reap the benefits long term), investors (as new systems would be required less frequently), and the environment more widely (due to decreased waste of materials). The production of sustainable products is also, arguably, of benefit to those selling robotic systems, as studies suggest that consumers are becoming more socially and environmentally conscious in their purchase decisions (Bucic et al., 2012; Lee and Rim, 2018).

### Pepper the Robot as More than an Object

In this study, some participants maintained the stance that Pepper was simply “an object”, “a machine”, and “a thing” whereas others indicated that they perceived the robot as having gender, and being able to feel emotion and pain (n = 33). The differences in responding suggest that for some, a social robot could be considered as a social entity, whereas for others, it could be perceived as simply an appliance or other product. Such findings contradict research which suggests that “humans have a natural tendency to anthropomorphize” (Reeves and Nass, 1996; Nie et al., 2012) - a statement which implies that the attribution of human-like qualities occurs to the same extent, and with the same time course, for all people. It could be the case that all humans do anthropomorphize machines in the same way, and that the participants were being deceptive in their responses for some reason, however we have a tendency to reject this idea due to work demonstrating differences on questionnaires probing general anthropomorphism (Chin et al., 2004; Waytz et al., 2010), and the range of individual differences that exist in so many other aspects of cognition (Lee and Webb, 2005; Gruszka et al., 2010; Parasuraman and Jiang, 2012).

Other research suggests that individual differences are not the only factor influencing how a robot is perceived. It has been found that the perception of a robot as having qualities of a ‘living’ agent (e.g., intentions, emotions, and the ability to feel hurt) is sensitive to numerous factors including (but not limited to) the appearance of the robot (e.g., humanoid vs non-humanoid), the robot’s behavior (e.g., congruent vs incongruent gesture and speech), and the beliefs of the participant about the origin of the behavior (e.g., an algorithm vs human-controlled) (Salem et al., 2013; Wykowska et al., 2015; Hortensius and Cross, 2018; Crowell et al., 2019). If robots were perceived in a stable manner across people we could use existing models of attachment (Ball and Tasaki, 1992; Mugge et al., 2005) to predict the trajectory of robot uptake and usage, however the studies and insights outlined previously suggest that such fixed attitudes are not yet a reality. It could be suggested that if a robot were deployed in a certain setting, and programmed to behave in a consistent way, that there could be wide scale acceptance and usage, but without an appreciation for individual differences and changes in attitudes over time, we argue that this is unlikely to be the case. Despite being a complicated venture, a better understanding how humans perceive robots, and how these feelings evolve across time (e.g., Riddoch and Cross, 2020) is becoming increasingly important, as in some instances, it could be inappropriate/harmful to for humans to feel attached to robots - e.g., military personnel with bomb-disposal robots, or vulnerable people with care robots (Sharkey and Sharkey, 2012; Darling, 2015; Lin et al., 2017).

### Methodological Reflections

In order to probe phenomena such as robot abuse and obedience, the need to maintain realism and minimize participant stress must be balanced (Geiskkovitch et al., 2016). Geiskkovitch and colleagues (2016) provide brief guidelines for researchers hoping to do so, and include the following recommendations: 1) Participants can leave at any time; 2) Immediate and thorough debriefing; 3) Reflection time; 4) Contingency plan; 5) Participant safety and comfort; and 6) Effect on researchers. The latter point is important to emphasize as researcher wellbeing is often neglected in HRI work, despite evidence that it can be negatively impacted by involvement in confrontational/awkward/unsettling social with participants and robots scenarios (Rea et al., 2017; Geiskkovitch et al., 2015, & Young, 2017). In addition to the guidelines presented by Geiskkovitch and colleagues (2016), we might add that a fuller understanding of the challenges and opportunities presented by studying human moral behavior with robots is likely to be achieved if experimenters take the time to revisit and read the major codes of ethics that govern research with human participants, such as the Declaration of Helsinki (1964; 1996), the Belmont Report (1979), and the British Psychological Society (2014) Code of Human Research Ethics. Research in social robotics and human-robot interaction continues to rapidly develop. It will consequently be vital for researchers working on the front lines of this discipline to actively reflect on and refine ethical guidelines, based on the hallmark ethical treatizes on research with human participants that have come before, as well as responding to new ethical challenges that research with robots presents.

We conducted this study in a research laboratory, controlling the interaction between the participant and the robot, and yet we observed large variation in participant responses and attachment to the robot. In real-world environments, human-robot interactions will likely be subject to many more influences, providing a multi-layered and dynamic challenge for researchers and industries hoping to introduce socially assistive robots at scale. The types of contextual and individual differences that will shape human-robot interactions range from media exposure and novelty (two themes touched on here) to the environment the robot is situated in, participant expectations, ongoing experience, age, and many more besides (Cross & Ramsey, under review). While we only considered one HRI scenario in the present study, we hope that the insights gathered here will inform future work across a range of different settings, by highlighting the range of responses and perceptions that underpin participants’ observable behavior toward robots.

### Open Ended Questions Add Research Value

This study, and the rich insights it has yielded about the variety of reasons people hesitate or comply with instructions to harm a friendly humanoid robot, lead us to add our voices to those strongly advocating for greater inclusion of social scientists in future human–robot interaction research (Broadbent, 2017; Meiji and Kajikawa, 2017; Irfan et al., 2018; Henschel et al., 2020), as well as methods that adopt an open approach to post-experiment questioning. By adopting an open approach to questioning (e.g., “What do you think about the robot?”), compared to closed questions/scales/continua, it is possible to gain deeper and clearer insight into the respondents’ experience or knowledge (Mathers et al., 1998; Copeland, 2017; Kara, 2018). While the data and insights yielded by such open-ended approaches are certainly messier and less structured compared to many other kinds of dependent variables measured in experimental psychology (for example), they also hold great potential facilitating innovative new research questions that might be missed if researchers are reluctant to stray from tried-and-true questionnaires and short-answer debriefing procedures.

In this study, the use of open questions allowed us not only to gain a better understanding of some of the factors affecting hesitation, but also peoples’ thought processes, their feelings of cognitive overload and confusion, perceived power imbalances between themselves and the robot, and individual differences in anthropomorphism and feelings of friendship and connection toward the robot. Without such questions, it is likely that such insights would have been missed and we advocate for greater use of mixed-methods experiments in human-robot interaction research as a result. Even in cases where experimenters measure reaction times or other seemingly ‘objective’ responses, we argue that it is still valuable to provide participants with an opportunity to openly provide feedback about their experience. It is possible that they used a strategy which affected responding, or they perceived the stimuli differently to how researchers might have expected. Collecting such data may lead to complexities when trying to interpret data, but we would argue that this intricacy reflects a greater understanding of the phenomenon and measurement techniques, and is a valuable use of resources as a result. While this approach could be valuable for all manner of behavioral psychological experiments, we would argue it is especially vital for experiments examining human-robot interaction. Because so many unknowns still exist concerning the human side of human-robot interaction, a more nuanced understanding of people’s thoughts and feelings beyond the confines of strict experimental instructions can help shape and advance this new and burgeoning field in a way that improves the utility of robots for human users (c.f. Cross et al., 2019).

As past work has convincingly argued, although words can be insightful and thought-provoking, they are susceptible to factors such as experimenter bias and social desirability effects (Gilder and Heerey, 2018; Bergen and Labonte, 2020). Such factors lead to an increased possibility that the participants’ responses do not affect their true thoughts and feelings. By investigating the question with a combination of methodological approaches (e.g., behavioral and qualitative), using double-blinding procedures, following guidelines regarding experimenter behavior and demeanor (Bergen and Labonte, 2020), and by making a conscious effort to unearth and negate experimenter biases (Nowell et al., 2017), it is possible to strengthen the validity of participant claims (Gibson, 2017).

While no universally agreed-upon framework for ensuring scientific rigor when using qualitative approaches exists yet, a number of articles from social and health sciences detail how a researcher can improve the rigor of their own qualitative research practice (Braun and Clarke, 2006; Nowell et al., 2017; Sundler et al., 2019). Although not specific to the field of HRI, such articles are insightful as they detail practical recommendations for how to improve validity, rigor, credibility and transparency of qualitative research in general. For example, some authors propose using methods such as journaling thought-processes, explicitly stating potential biases in publications, and the use of independent coders (Nowell et al., 2017; Sundler et al., 2019). Such methods are not restricted to a specific field of study and, thus, are as applicable to HRI as any other behavioral experimental field as a result. A number of books detailing practical advice for undertaking mixed-methods research - investigation using both quantitative and qualitative approaches – also exist and serve as useful resources for researchers wishing to incorporate these approaches (e.g., Bernard, 2013; Brannen, 2017). To streamline methods, know which common pitfalls to avoid, and educate upcoming researchers in HRI, it would benefit the field to curate resources providing subject-specific examples.

In addition to assisting with the validation of measurement techniques, the results of qualitative analysis could generate new research questions and bridge interdisciplinary communications - e.g., between roboticists, computer scientists, and social scientists (to name a few). The ‘Open Science’ movement means that more resources are accessible for free, however specialist language continues to be a barrier for understanding and collaboration (Salgian et al., 2011). By using the words of the general public to compliment quantitative experimental data, this could help to bridge interdisciplinary barriers. In addition to promoting collaboration between specialties, the inclusion of such quotes and thoughtful discussion could make scientific content more accessible and engaging for the general public, and young people interested in STEM professions. Such accounts could also incite innovative public engagement activities, and inspire narratives for books, games, and other creative ventures.

## Conclusion

In this study we demonstrate that the participants’ insights on their thoughts and feelings toward social interactions with robots can be incredibly useful in human-robot interaction experiments designed to probe prosocial attitudes and behaviors toward robots - for the validation of measures, but also a broader understanding of how tasks effect and influence participants. They also allow for insight into the topic of interest more broadly, and can allow for the generation of exciting new research questions and approaches. It is possible that quotes and discussions could also bridge the barrier between specialists, the general public, and those working on creative projects to engage and inspire.

## Data Availability

The datasets analysed for this study are available on the following Open Science Framework page: https://osf.io/d7c8t/.
